# Anti-Aβ Oligomer IgG and Surface Sialic Acid in Intravenous Immunoglobulin: Measurement and Correlation with Clinical Outcomes in Alzheimer’s Disease Treatment

**DOI:** 10.1371/journal.pone.0120420

**Published:** 2015-03-31

**Authors:** Hyewon Kwon, Amanda C. Crisostomo, Hayley Marie Smalls, John M. Finke

**Affiliations:** 1 Department of Medicinal Chemistry, University of Washington, Seattle, Washington, United States of America; 2 Division of Science and Mathematics, University of Washington, Tacoma, Washington, United States of America; Weizmann Institute of Science, ISRAEL

## Abstract

The fraction of IgG antibodies with anti-oligomeric Aβ affinity and surface sialic acid was compared between Octagam and Gammagard intravenous immunoglobulin (IVIG) using two complementary surface plasmon resonance methods. These comparisons were performed to identify if an elevated fraction existed in Gammagard, which reported small putative benefits in a recent Phase III clinical trial for Alzheimer’s Disease. The fraction of anti-oligomeric Aβ IgG was found to be higher in Octagam, for which no cognitive benefits were reported. The fraction and location of surface-accessible sialic acid in the Fab domain was found to be similar between Gammagard and Octagam. These findings indicate that anti-oligomeric Aβ IgG and total surface sialic acid alone cannot account for reported clinical differences in the two IVIG products. A combined analysis of sialic acid in anti-oligomeric Aβ IgG did reveal a notable finding that this subgroup exhibited a high degree of surface sialic acid lacking the conventional α2,6 linkage. These results demonstrate that the IVIG antibodies used to engage oligomeric Aβ in both Gammagard and Octagam clinical trials did not possess α2,6-linked surface sialic acid at the time of administration. Anti-oligomeric Aβ IgG with α2,6 linkages remains untested as an AD treatment.

## Introduction

Alzheimer’s Disease (AD) cases and associated health care costs are growing at a faster rate than most other diseases due to an aging population and lack of effective treatment [1]. Based on success in early pre-clinical studies, a number of recent antibody treatments offered hope for AD patients [2–9]. Unfortunately, none of these treatments met their primary endpoints in Phase II and III clinical trials [2–5]. While disappointing, these results do provide an opportunity to learn what is missing and how newer formulations might yield success. Given that multiple antibodies have now been tested in AD patients, one can compare properties between the antibody treatments which reported small benefits from those with no cognitive benefit [2–5]. Also, some antibody drugs failed AD clinical trials despite their success in treating other diseases [2–5]. This class of AD drug offers a unique opportunity to determine why their biological activity did not improve AD patient outcomes.

Due to its complex polyclonal nature, intravenous immunoglobulin (IVIG) is an AD drug candidate that provides a system in which multiple properties can be investigated. Until recently, IVIG was a leading candidate for AD treatment based on small early trials that indicated a possible reduction of AD progression [6,7,10,11]. Octagam (OCT) and Gammagard (GG) are the most rigorously tested IVIG products for AD treatment [2,3,12]. Two Phase II trials with Octagam and a Phase III trial with Gammagard have been completed [2,3,12]. A Phase III trial of a third IVIG product, Flebogamma (Grifols), is currently underway but results are not available [13].

A biochemical analysis and comparison of Octagam and Gammagard is warranted at this time because slightly different outcomes are reported from their clinical trials. Octagam lacked any clinical efficacy while Gammagard was reported to slow AD progession in the moderate AD and ApoE4 carrier subgroups [2,3]. The effect of Gammagard was small and the statistical significance was marginal because the study was not powered for subgroup analysis. Nonetheless, biochemical differences between these Octagam and Gammagard may provide clues about how to enhance the efficacy of AD antibody therapies.

In addition, Octagam and Gammagard are both approved for use in treating a number of autoimmune disorders [14–16]. Neuroinflammation is associated with AD but its role in the pathology has not been definitively established [17]. Investigations of putative anti-inflammatory components within IVIG might reveal how the success of IVIG with autoimmune conditions can be translated into benefits for AD patients.

A number of components and properties exist in Octagam and Gammagard that one could compare. However, two general properties are logical to investigate at this stage. The first is IgG specific for Aβ soluble oligomers (OAβ). The second is the sialic acid moieties of IgG.

Interest in IVIG as an AD treatment was initially based on a significant fraction of antibodies in IVIG that bound to Aβ peptides [10,18]. An ELISA study has shown that Octagam has a higher fraction of IgG that bind the Aβ peptide than Gammagard [19]. However, this comparison has not been made using specific preparations of OAβ as the target ligand. OAβ is more causally linked to AD pathology than either the monomeric or fibrillar form of Aβ [20–22]. Also, a previous SPR study indicates that monomeric Aβ at the sensor surface is not bound by antibodies in IVIG [23]. The ELISA studies that detect natural IgG binding to monomeric Aβ consistently report a lower binding response than to OAβ [24–27]. Thus, OAβ was the preferred Aβ assembly state to capture natural IVIG antibodies in the present study.

Glycan sialic acid in IgG was also of interest because higher levels of IgG sialic acid are reported to be associated with increased anti-inflammatory activity of IVIG in select animal models and in clinical studies with patients [16,28,29]. The level of sialic acid in therapeutic antibodies has not been generally reported in the context of AD treatment. However, a component of antibodies that might reduce neuroinflammation could be beneficial in AD treatment [17]. Thus, investigation of IgG sialic acid in the present study was warranted.

For Octagam and Gammagard, we report here the relative fraction of anti-OAβ IgG (OAβ^+^ IgG), the fraction of surface-accessible sialic acid on IgG, the IgG domain where the surface sialic acid predominates, and the fraction of surface sialic acid in the anti-OAβ IgG subpopulation. All variables are assessed for their correlation with the putative clinical efficacy of Gammagard. The surface sialic acid of mouse monoclonal antibodies 6E10 and 4G8 are also presented for comparison because these two anti-Aβ antibodies exhibit distinct glycan sialylation levels and localization.

All quantitative measurements were conducted using two advanced applications of Biacore Surface Plasmon Resonance (SPR). The first method was Calibration-Free Concentration Analysis (CFCA), a method that quantifies the concentration of bulk analyte in a solution under conditions where analyte association with the sensor surface is diffusion-limited [30–33]. The second method is a two-step method, in which IgG is bound to the sensor ligand in the first step and sialic acid is detected using *Sambucus nigra* Agglutinin (SNA) or other carbohydrate-specific lectins in the second step.

While SPR label-free methods are emerging as useful research tools [34,35], the two SPR methods used here are novel approaches in the quantitative measurement of OAβ^+^ IgG and sialylated fractions in IgG preparations. From a technical standpoint, SPR is advantageous because it minimizes complications from nonspecific background antibody interactions that have been problematic in similar studies [24,36,37]. The low SPR background is the result of a brief IgG binding step and chemically inert sensor surface [23]. Background signal that does exist is readily identified in the reference flow cell and corrected in real time.

From a scientific standpoint, the present SPR methods facilitate the direct measurement of IgG with “surface” sialic acids that are capable of direct binding with other biomolecules. For purposes of the present study, we further specify this IgG subgroup as SNA^+^ IgG, defined by its capacity to bind SNA lectin [38]. A number of prior studies have investigated total sialic acid, Fc glycan sialylation, or Fab N-glycan sialylation in IVIG using SNA lectin affinity chromatography, HPLC and/or Mass Spectrometry [16,28,29,39–42]. These studies have demonstrated that different sialic acid groups in IgG do not contribute equally to SNA binding [29,39,40]. While these experiments have provided valuable insights, important questions remain that are relevant to AD treatment. First, the endogenous SNA^+^ IgG fraction of IVIG has not been directly measured as this property has thus far been inferred from affinity chromatography yields [29,39,40]. Second, the maximum possible SNA^+^ IgG fraction after enzymatic sialylation has not been determined. Third, the SNA-binding subfraction of OAβ^+^ IgG has not been determined.

Here, SPR is used to directly measure the concentration of OAβ IgG subfraction, SNA^+^ IgG subfraction, and the combined SNA OAβ^+^ IgG sub-subfraction in IVIG. This study is conducted on Octagam and Gammagard, two IVIG products with different clinical outcomes in AD clinical trials. Treatment with α2,6-sialyltransferase (2,6ST) and neuraminidase (NEU) are used to determine the respective maximum and minimum SNA^+^ IgG fraction possible after enzymatic treatment. The SNA-binding contribution from Fab sialic acids in IgG is determined directly using EndoS enzymatic treatment to remove the Fc glycan from intact IgG preparations. For comparison, these properties are also measured in anti-Aβ monoclonal antibodies 6E10 and 4G8.

This study is important because we do not fully understand the biological activity resulting from Aβ-specific IgG, sialylated IgG, and IgG sialylation at different Fab and Fc sites. While the work focuses on IVIG products used in AD treatment, the findings complement a broader range of research efforts on IVIG treatment of autoimmune diseases [16,28,29,39–41]. Taken together, it is hoped that these findings will help identify new formulations of therapeutic antibodies that yield better treatment outcomes for AD patients.

## Materials and Methods

### Materials

IVIG product 5% Octagam Lot A322B8431 (OCT) was from Octapharma (Vienna, Austria) and 10% Gammagard Liquid LE12M126AB (GG) was from Baxter (Los Angeles, CA). Monoclonal antibodies 6E10 and 4G8 were purchased from Covance. Aβ_1–42_ peptide was purchased from Anaspec (San Jose, CA). Two lots of 4G8 were studied and are referenced as 4G8a and 4G8b. Unconjugated and biotinylated lectin *Sambucus nigra* agglutinin (SNA) and unconjugated *Erythrina crystalgalli* lectin (ECL) was obtained from Vector Laboratories (Burlingame, CA). Protein A from *Staphylococcus aureus* (SpA), α-2,6 sialyltransferase from *Photobacterium damsela* (2,6ST), Cytidine-5′-monophospho-N-acetylneuraminic acid (CNA) and PNGase F from *Chryseobacterium meningoseptica* were obtained from Sigma-Aldrich (St. Louis, MO). Recombinant neuraminidase from *Clostridium perfringens* (NEU) was purchased from New England Biolabs (Ipswich, MA). EndoS enzyme IgGZERO was obtained from Genovis AB (Lund, Sweden). Biacore running buffer containing 10 mM HEPES, 150 mM NaCl, 3 mM EDTA, 0.005% v/v, pH 7.4 (HBS-EP) was obtained from a 10X stock (GE Life Sciences).

### IgG Enzyme Treatments

The fraction of SNA^+^ IgG was increased to its maximal level using α2,6ST treatment consisting of 5 μL IgG solution containing 5 μg (33 pmol) mAb or 50–500 μg (0.3–3.3 nmol) IVIG, 5 μL of 1 M Tris pH 8 reaction buffer, 5 μL of 15 mM CNA (75 nmol), 1 μL 2,6ST (25 milliunits), and 34 μL water. Sialic acid was removed from IgG antibodies using NEU treatment consisting of a 5 μL IgG solution containing either 5 μg mAb (33 pmol) or 50–500 μg (0.3–3.3 nmol) IVIG, 5 μL 10X reaction buffer (500 mM citrate, pH 6), 3 μL NEU (150 Units), and 37 μL water. Fc glycans were removed from IgG antibodies using EndoS treatment consisting of a 4 μL IgG solution containing either 4 μg (33 pmol) mAb or 4–150 μg (0.3–1.2 nmol) IVIG, 32 μL phosphate buffered saline pH 7.4 (PBS), and 4 μL IgGZERO containing 80 Units of EndoS enzyme.

All N-linked glycans were removed from denatured IgG using a two-step procedure. The first step involved complete denaturation of 4 μg mAb or 40 μg IVIG with 44 μL of 0.2% SDS, 100 mM β-mercaptoethanol, 50 mM phosphate, pH 7.5 for 100° C. The second step involved addition of 4 μL of either 15% Triton X-100 or 4 μL 10% NP-40 followed by 500 mU (Sigma Units) of PNGase F.

All enzyme treatments involved incubation at 37°C for a minimum of 3 hours, with overnight incubation if possible. After enzymatic treatment, the IgG were dialyzed into HBS-EP Biacore running buffer and studied within 24 hours after removal from HBS-EP dialysis.

### Aβ Oligomer Preparation

Aβ_1–42_ oligomers (OAβ) were produced at high concentration to produce high density SPR sensor chips suitable for CFCA. Briefly, disaggregation of purchased Aβ_1–42_ was ensured by dissolution in 50:50 trifluoroacetic acid (TFA):hexafluoroisopropanol (HFIP) at a 1 mg:1 ml peptide:solvent ratio, bath sonicated for 1 h at room temperature, and evaporated to dryness with a gentle stream of Argon gas [43]. The disaggregated Aβ_1–42_ was resuspended at 20 mg/ml in dimethylsulfoxide (DMSO) and diluted 1/40 with a solution of 100 mM phosphate and 100 mM NaCl, pH 7.4, resulting in an expected concentration of 114 μM Aβ_1–42_ [44]. This solution was centrifuged at 14,000 x g for 10 min and filtered through a 10,000 MWCO Amicon filter. The soluble concentration in this preparation was 104 μM, determined quantitatively using the extinction coefficient of tyrosine 10 [45]. This preparation produced a high concentration of oligomers that reached a maximal concentration after 2–3 weeks at room temperature, at which point > 95% of monomers had reacted into an assembly state. While some loss into insoluble aggregates did occur over a period of months, the soluble oligomer concentration remained high at > 50% of total Aβ_1–42_ if the sample was left unperturbed.

It is acknowledged that soluble oligomer shapes, sizes and molecular conformations can vary with preparation conditions [46–52]. In some studies, considerable effort has been devoted to producing a desired oligomer state at high purity [50]. For the present CFCA experiments, the objective is to produce and immobilize enough high-affinity antigenic oligomers so that IgG binds the sensor chip under mass transport limited conditions [30]. If these conditions are established, complications from any immobilized low-affinity oligomer states are minimal because they do not contribute significantly to binding IgG during CFCA [31].

### SPR Sensor Chip Preparation and Regeneration

All SPR chip preparation and subsequent studies were performed using either a Biacore T100 or T200 system using Series S CM5 sensor chips (GE Healthcare). To ensure a representative background subtraction, the sample flow cell containing immobilized ligand was always placed immediately after a reference flow cell with no ligand. CM5 carxboxyl group activation was performed using 7 min of a 1:1 mix of 50 mM N-hydroxysuccinimide (NHS) and 0.2 M N-ethyl-N-dimethylaminopropylcarbodiimide (EDC). After ligand immobilization, remaining activated groups were capped using 1.0 M ethanolamine (pH 8.5) for 7 min. The immobilization reaction was also performed in the reference cell but with no ligand added.

For SpA flow cells, immobilization of 3649 RU was achieved using 0.1 mg/ml (2.3 μM) SpA in 10 mM sodium acetate pH 4.5 with an automated software routine (target 3500 RU). For SNA flow cells, immobilization of 8906 RU was achieved using 0.2 mg/ml (1.4 μM) SNA in 10 mM sodium acetate pH 4 with an automated software routine (target 12,000 RU). For OAβ flow cells, OAβ described in *A*β *Oligomer Preparation* was first centrifuged at 12000xg, supernatant dialyzed into 10 mM acetate pH 4, and re-centrifuged immediately prior to a 2 hour immobilization at 5 μL/min. This procedure produced 3266 of initially immobilized “native” OAβ. To facilitate routine SDS regeneration of the OAβ sensor chip with high affinity antibodies, the native OAβ was treated exhaustively with 0.5% SDS regeneration buffer resulting in 2305 RU of “SDS-resistant” OAβ. This SDS-resistant OAβ proved to be highly stable in repeated SDS regenerations from multiple IgG binding experiments.

After each IgG binding measurement, flow cell regeneration was performed using two 20 s pulses of 10 mM glycine pH 1.5 for immobilized SpA and two 20 s pulses of 10 mM HCl for immobilized SNA. Flow cells containing immobilized OAβ were regenerated with three 30 s pulses of 0.5% SDS after IVIG binding and regenerated with eight 30 s pulses plus three 60 s pulses of 0.5% after 6E10 or 4G8 binding.

### Calibration-Free Concentration Analysis

**Fig 1:**
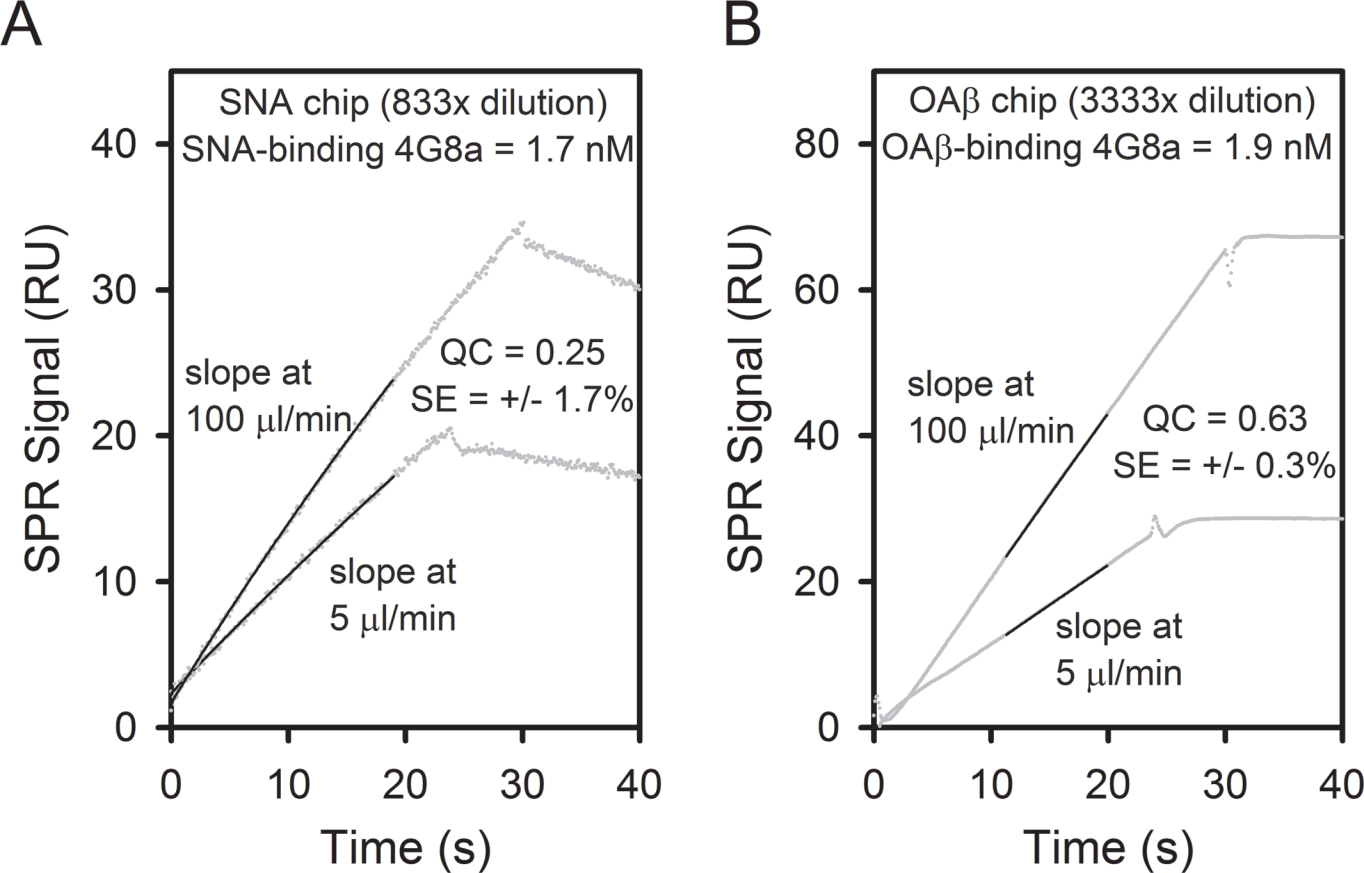
CFCA measurements of IgG subgroups with high ligand affinity. CFCA raw SPR data and fits of 4G8a association using a 833-fold stock dilution in a flow cell with immobilized SNA lectin (A) and a 3333-fold stock dilution in a flow cell with immobilized OAβ (B). The raw data and fits at flow rates 5 μl/min and 100 μl/min are indicated along with the QC parameter and percent standard error SE of the CFCA fit. The concentration of the original 4G8a stock determined from UV measurements was 6500 nM.

CFCA was performed using two sequential injections of IgG at different flow rates, 5 and 100 μl/min, and the association slope measured for 30 s under each flow rate. In general, only the middle time points of association period (5–25 s) were used in determining the slope to avoid signal artifacts at the start and end of the injection period. Representative CFCA measurements and fitted linear slopes are shown for mAb 4G8a on the SNA sensor chip (Fig 1A) and on the OAβ sensor chip (Fig 1B).

Using CFCA, the concentration of analyte in bulk solution [A_bulk_] with affinity for the immobilized ligand can be determined using Eq. 1 using two SPR association slopes (RU/s), m_1_ for low flow and m_2_ for high flow [30,33,34].

$$\begin{array}{ccc} \left[{\text{A}}_{\text{bulk}}\right] & = & \left[\frac{1.47\cdot \text{α}}{\text{G}\cdot \text{MW}}\right] \end{array}{\left[\frac{{\text{D}}^{2}}{{\text{h}}^{2}\text{B}}\right]}^{1/3}\left[\frac{\begin{array}{ccc} \frac{1}{{\text{F}}_{1}} & - & \frac{1}{{\text{F}}_{2}} \end{array}}{\begin{array}{ccc} \frac{1}{{\text{m}}_{1}} & - & \frac{1}{{\text{m}}_{2}} \end{array}}\right]$$ (1)

All variables in Eq. 1 other than m_1_ and m_2_ are known for a given experiment. G is the SPR response factor for sensor surface (10^6^ RU m^2^/g for CM5 sensor chips), MW is the molecular weight of the analyte (150,000 g/mol for IgG), D is the diffusion constant of the analyte (3.99 x 10^–11^ m^2^/s for IgG), h is the height of the flow cell (4.0 x 10^–5^ m for the Biacore T100), B is the width x length area of the flow cell (9.85 x 10^–6^ m^2^ for Biacore T100 flow cell 1), F_1_ is the lower flow rate (5 μl/min = 8.3 x 10^–11^ m^3^/s), F_2_ is the higher flow rate (100 μl/min = 1.7 x 10^–9^ m^3^/s), and α is a conversion factor from m^3^ to L (0.001 m^3^/L).

When all variable values except m_1_ and m_2_ are known or assumed for IgG in Eq. 1, the formula for [A_bulk_] = [IgG_bulk_] reduces a much simpler equation shown in Eq. 2.

$$\begin{array}{ccc} \left[{\text{IgG}}_{\text{bulk}}\right] & = & \frac{\text{β}}{\begin{array}{ccc} \frac{1}{{\text{m}}_{1}} & - & \frac{1}{{\text{m}}_{2}} \end{array}} \end{array}$$ (2)

In Eq. 2, the coefficient β = 6.5 x 10^–10^ (mol*s)/(L*RU) incorporates all known variables of IgG properties and the Biacore T100 flow cell 4 in Eq. 1. For flow cells with different value of h or B in either the T100 or T200, the system software automatically incorporates these dimensional changes into the appropriate value of β.

CFCA provides the bulk solution concentration of IgG species with affinity for the ligand in the flow cell [IgG_bulk_]. The SNA flow cell therefore provides the concentration of IgG with at least one glycan exhibiting a surface accessible α2,6-galactose-sialic acid linkage, denoted here as [IgG]_FC_SNA_ [38]. The SpA flow cell provides a measurement of >95% of human IgG, with the CFCA concentration denoted here as [IgG]_FC_SpA_ [53,54]. The OAβ flow cell provides the concentration of OAβ^+^ IgG, denoted here as [IgG]_FC_OAβ_.

Ratios of these IgG subpopulations shown in Eq. 3A, 3B and 3C below provide the fraction of OAβ^+^ IgG in IVIG (F_OAβ+ IVIG_), SNA-binding IgG in IVIG (F_SNA+ IVIG_), and SNA-binding IgG in mAbs 4G8 and 6E10 (F_SNA+ mAb_).

$$\begin{array}{ccc} {\text{F}}_{\text{OAβ+IVIG}} & = & \frac{{\text{X}}_{\text{FC\_OAβ}}*{\left[\text{IgG}\right]}_{\text{FC\_OAβ}}}{{\text{X}}_{\text{FC\_SpA}}*{\left[\text{IgG}\right]}_{\text{FC\_SpA}}} \end{array}$$ (3A)

$$\begin{array}{ccc} {\text{F}}_{\text{SNA+IVIG}} & = & \frac{{\text{X}}_{\text{FC\_SNA}}*{\left[\text{IgG}\right]}_{\text{FC\_SNA}}}{{\text{X}}_{\text{FC\_SpA}}*{\left[\text{IgG}\right]}_{\text{FC\_SpA}}} \end{array}$$ (3B)

$$\begin{array}{ccc} {\text{F}}_{\text{SNA+mAb}} & = & \frac{{\text{X}}_{\text{FC\_SNA}}*{\left[\text{IgG}\right]}_{\text{FC\_SNA}}}{{\text{X}}_{\text{FC\_OAβ}}*{\left[\text{IgG}\right]}_{\text{FC\_OAβ}}} \end{array}$$ (3C)

In Eq. 3A, 3B and 3C, X_FC_SpA,_ X_FC_OAβ,_ and X_FC_SNA_ refer to associated multiplication factors that account for any dilution of the original stock IgG solutions. For brevity, the term F_SNA+IgG_ in the present article is used to refer generally to F_SNA+IVIG_ and F_SNA+mAb_ collectively or individually.

The validity of a given [IgG] measurement by CFCA was determined by fulfilling two criteria. The first is that the standard error of the CFCA fit must be less than 20% of the reported [IgG] concentration. The second is that the QC parameter shown in Eq. 4 exceeds 0.1 [30,31].

$$\begin{array}{ccc} \text{QC} & = & \frac{\begin{array}{ccc} \frac{{\text{m}}_{2}}{{\text{m}}_{1}} & - & 1 \end{array}}{\begin{array}{ccc} {\left(\frac{{\text{F}}_{2}}{{\text{F}}_{1}}\right)}^{1/3} & - & 1 \end{array}} \end{array}$$ (4)

QC in Eq. 4 determines the degree to which the interaction of ligand-binding IgG with the immobilized ligand is diffusion limited (i.e. mass transport limited). A partial and detectable degree of mass transport limitation is a necessary criteria for CFCA [30,31]. In theory, QC varies between 0 (no diffusion limitation) to 1 (complete diffusion limitation). While the Biacore manual suggests QC > 0.2 for CFCA, we have found that our CFCA measurements retained reliable accuracy down to QC ~ 0.1 provided the percent standard error of the fit was < 20%. Fig 1A and 1B show CFCA results that met the criteria using 4G8a.

Except where noted, all reported values determined from CFCA exceeded the threshold of QC > 0.1 and had a standard error less than 20% of the reported value. The QC ratio for IgG measurements of the SNA flow cell ranged between 0.2–0.3. The QC ratio for mAb binding in the OAβ flow cell ranged between 0.4–0.8. The QC ratio for IVIG binding to the SpA flow cell ranged between 0.3–0.5. The QC ratio for OAβ^+^ IgG in IVIG binding to the OAβ flow cell ranged between 0.2–0.4. These QC ratios were achieved using working dilutions of the original IgG stock solution as follows: Gammagard (10%): X_FC_OAβ_ = 100, X_FC_SNA_ = X_FC_SpA_ = 10,000; Octagam (5%): X_FC_OAβ_ = 50, X_FC_SNA_ = X_FC_SpA_ = 5,000; 6E10/4G8 mAbs (1 mg/ml): X_FC_OAβ_ = X_FC_SpA_ = 3,000, X_FC_SNA_ = 1000.

The accuracy of CFCA was also validated using 3333-fold dilutions of 6E10 and 4G8a stocks, confirmed to be 6.7 and 6.5 μM respectively by UV spectroscopy, with working test concentrations of 2.0 nM mAb. The CFCA concentration of 6E10 was determined at [IgG]_FC_OAβ_ = [IgG]_FC_SpA_ = 1.8 nM using both the OAβ sensor chip and the Protein A sensor chip. The CFCA concentration of 4G8 was determined at [IgG]_FC_OAβ_ = 1.9 nM using the OAβ sensor chip (Fig 1B) and [IgG]_FC_SpA_ = 2.0 nM using the Protein A sensor chip. As all of these values fell within 10% of the expected value of 2.0 nM, the accuracy of the CFCA method on IgG with Protein A and OAβ sensor chips was established. Unfortunately, a reliable IgG standard for the SNA chip is not available. Given the success with Protein A and OAβ sensor chips, the accuracy of the SNA chip with CFCA was assumed to be reasonable provided the two aforementioned CFCA criteria were met.

### On-Chip Analysis

**Fig 2:**
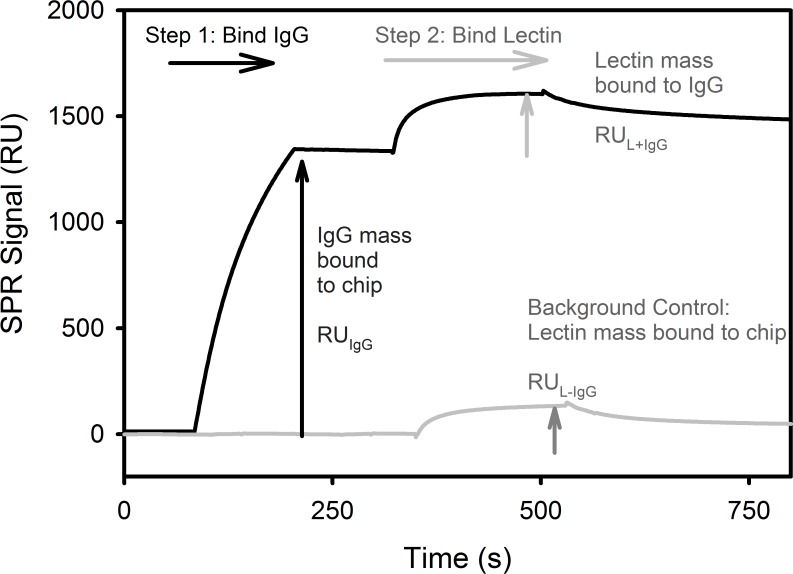
On-Chip SPR measurement of IgG surface sialic acid. A representative SPR On-Chip experiment of a 66-fold dilution of 4G8a in a flow cell with immobilized OAβ. In Step 1, IgG (4G8a) is bound for 120 s and the association binding signal, RU_IgG_, is measured. In Step 2, 3 μM SNA lectin is bound for 180 s to the flow cell containing IgG and this second association signal, RU_L+IgG_, is measured. The background binding signal of 3 μM SNA, RU_L-IgG_, is measured for the flow cell in the absence of IgG (grey curve/arrow).

As a complementary method to determine F_SNA+ IgG_, an ELISA-based “On-Chip” technique was also used to determine F_SNA+ IgG_. The On-Chip method was also used to determine F_ECL+IgG,_ the fraction of IgG that bound ECL, a lectin specific to terminal galactose residues with an α1,4 linkage to a preceding N-aceytylglucosamine [55]. The On-Chip method involves an initial binding step of IgG followed with a second binding step with excess SNA (3 μM) or excess ECL (33 μM). A representative example of F_SNA+IgG_ measurement of 4G8a in the OAβ flow cell is shown in Fig 2. All On-Chip F_SNA+ IgG_ measurements for 6E10 and 4G8 were performed using the OAβ flow cell. For IVIG, the SpA flow cell was used to determine F_SNA+ IgG_ for total IVIG and the OAβ flow cell was used to determine F_SNA+ IgG_ of the OAβ^+^ IgG subgroup.

Regardless of whether the immobilized ligand in the flow cell was SpA or OAβ or whether the initial IgG measured was IVIG or mAb, the fraction of SNA or ECL Lectin-binding IgG (F_L+IgG_) was determined using Eq. 5.

$$\begin{array}{ccc} {\text{F}}_{\text{L}+\text{IgG}} & = & \frac{\text{M}*\left(\begin{array}{ccc} {\text{RU}}_{\text{L}+\text{IgG}} & - & {\text{RU}}_{\text{L}-\text{IgG}} \end{array}\right)}{{\text{RU}}_{\text{IgG}}} \end{array}$$ (5)

In Eq. 5, RU_IgG_ is the SPR response units of the first binding step involving IgG (~1300 RU in Fig 2). RU_L+IgG_ is the SPR response units produced in the second binding of lectin with IgG bound to the sensor (~270 RU in Fig 2). RU_L-IgG_ is a background control on the flow cell without IgG bound (~130 RU in Fig 2) and is subtracted from RU_L+IgG_ to determine the true value of SNA binding (~140 RU). M is the mass ratio of 150 kD IgG to lectin L (1.07 for 140 kD SNA, 2.78 for 57 kD ECL) [38,55]. F_SNA+IgG_ denotes the value of F_L+IgG_ for SNA lectin and F_ECL+IgG_ denotes the value of F_L+IgG_ for ECL lectin.

The On-Chip method was also used to determine the extent to which EndoS enzyme reduces SNA-binding by selective cleavage of the Fc glycan. The susceptibility of F_SNA+IgG_ to EndoS treatment was represented by the variable F_EndoS_ in Eq. 6.

$$\begin{array}{ccc} {\text{F}}_{\text{EndoS}} & = & \frac{{\text{F}}_{\text{SNA}+\text{IgG}}(+\text{EndoS})}{{\text{F}}_{\text{SNA}+\text{IgG}}(-\text{EndoS})} \end{array}$$ (6)

In Eq. 6, F_SNA+IgG_(+EndoS) refers to F_SNA+IgG_ of an IgG sample pretreated with EndoS enzyme and F_SNA+IgG_(-EndoS) refers to F_SNA+IgG_ of an IgG sample with “mock” EndoS enzyme treatment.

The criteria of On-Chip analysis is that a sufficiently high initial IgG binding is achieved in Step 1 to enable a reliable determination of SNA binding in Step 2. We have determined this minimum threshold to be approximately 50 RU of IgG bound. However, in most cases, the value of RU_IgG_ ranged between 500–1500 RU depending on the IgG product and preparation prior to SPR analysis. High values of IgG binding were achieved using IgG solution concentrations in On-Chip analysis as follows: (1) 100 nM (15 μg/ml) 6E10 or 4G8 mAb, (2) 2.7 μM (400 μg/ml) IVIG using the SpA flow cell, and (3) 27 μM (4000 μg/ml) IVIG using the OAβ flow cell.

### SDS-PAGE and Lectin Blots

SDS-PAGE and Lectin Blots used 4–20% Tris-HCl Ready Gels (Bio-Rad). Antibody samples were mixed 1:1 with Laemmli Sample Buffer (Bio-Rad) with a loading concentration of 1% SDS, heated to 95°C for 2 minutes and electrophoresis performed with Tris-Glycine-SDS (0.1% SDS) running buffer on a MiniProtean II (BioRad). Kaleidescope prestained standards (BioRad 161–0324) were run in the end lanes. Standard SDS-PAGE gels were imaged used Coomassie Blue staining, followed by fixing and destain steps. For Lectin Blotting, proteins were transferred to Westran S PVDF membranes (Whatman) using an iBlot system (Invitrogen). The membrane was blocked using Carbohydrate-free blocking buffer (Vector Labs) followed by overnight incubation at 4°C with biotinylated SNA (100 μg/ml). After washing, the membrane was incubated for 1 hour at room temperature with Qdot 625 streptavidin conjugate (Invitrogen, A10196, 1:1000 dilution of 1 μM stock). Bands were visualized and imaged with a Gel Logic 100 Digital Imaging System (Kodak) using a Dark Reader Transilluminator (Clare Chemical).

### Statistics

All quantitative data measurements were performed either in duplicate (n = 2) or triplicate (n = 3) as indicated in the Figure legends. All error bars shown in the Figures are the standard deviation of a given set of replicated measurements. While many potential comparisons of data sets in the present work were possible, the following data set comparisons were considered most relevant: (A) The same IgG with different enzymatic treatment conditions and (B) Different IgG species with the same enzymatic treatment condition. Prior to more intenstive statistical analysis, single variable ANOVA was used on all data sets corresponding to comparison A or B to avoid Type I errors and confirm statistically significant differences. If the ANOVA p-value was less than 0.1, a two-tailed student’s t-test was performed on each data pair in all comparisons A and B to determine those with the greatest statistical significance and lowest p-values. The lowest p-values determined through this analysis are shown in Figures by letter and asterisk superscripts. All statistical analysis was performed using the statistical functions and ANOVA toolpack of Microsoft Excel 2010.

## Results

### CFCA and the On-Chip method are complementary approaches to quantify IgG subpopulations

Representative CFCA experiments using mAb 4G8 Lot A (4G8a) as the IgG species are shown in the flow cell with immobilized SNA lectin ligand (Fig 1A) and with immobilized OAβ ligand (Fig 1B). SPR slopes are shown under low flow at 5 μl/min and under high flow at 100 μl/min. Using Eq. 1 and Eq. 2 in Methods and Materials, these slopes are used to determine the bulk concentration of ligand-binding IgG in the solution passing through the flow cell [30]. In both Fig 1A and 1B, a mass transport limitation is qualitatively evident by the difference in the low and high slopes [31]. The use of CFCA is validated quantitatively by QC parameters > 0.1 (Eq. 4) and standard error SE < 20%.

Fig 1A shows immobilized SNA binding from a 833-fold dilution of 4G8a. Because only a fraction of antibodies exhibit surface sialic acid, the SNA-binding concentration is always less than the total IgG concentration [16,28,29,39–41]. Despite attempts to increase immobilized SNA the QC parameters for IgG binding in the SNA-flow cell were generally modest and ranged between 0.2–0.3.

Data in Fig 1B shows OAβ binding in a 3333-fold dilution of 4G8a. For anti-Ab mAbs such as 4G8 and 6E10, the data in Fig 1A will determine the total concentration of these mAbs assuming they are 100% active. If the solution measured in the OAβ-flow cell is serum IgG or IVIG, the determined concentration of OAβ^+^ IgG will be less than that of total IgG. The SPR association slopes in Fig 1B are highly linear and well separated, resulting in the high QC ratio of 0.63. In general, this was observed for all OAβ^+^ IgG binding from mAbs and IVIG, consistently yielding QC parameters between 0.5–0.8.

CFCA analysis shown in Fig 1A and 1B was used for IVIG and for anti-Aβ mAbs 6E10 and 4G8. CFCA was also performed using a flow cell with immobilized Protein A (SpA) to determine the post-preparation concentration of human IgG in the IVIG samples [54]. To compare Octagam, Gammagard, and OAβ^+^ mAb references, three fractional values were determined using Eq. 3A-3C (Methods and Materials). The fraction of OAβ^+^ IgG in IVIG (F_OAβ+IVIG_) was determined using Eq. 3A. The fraction of SNA^+^ IgG in IVIG (F_SNA+IVIG_) was determined using Eq. 3B. The fraction of SNA^+^ IgG in mAbs (F_SNA+mAb_) was determined using Eq. 3C. For clarity, the term F_SNA+IgG_ indicates either F_SNA+IVIG_ and/or F_SNA+mAb_.

Values of F_SNA+IgG_ from CFCA were also confirmed using the On-Chip method shown in Fig 2. For consistency with Fig 1, 4G8a is also used in Fig 2. The On-Chip method initially captures IgG using either immobilized SpA or OAβ in Step 1. The SPR signal produces a response RU_IgG_. After a brief equilibration period, excess SNA lectin (3 μM) is introduced in Step 2, producing a second binding response RU_L+IgG_ resulting from SNA binding to the IgG. To account for SNA binding to the sensor surface, a control without IgG is performed to determine the background response RU_L-IgG_. Once the sample and background control are measured, F_L+IgG_ is determined using Eq. 5 (Methods and Materials).

If SNA lectin was used, F_L+IgG_ is specified as F_SNA+IgG_ (Fig 2). A similar On-Chip method to that shown in Fig 2 was performed using 33 μM ECL lectin instead of 3 μM SNA. ECL ligand is used to quantify the fraction of IgG with surface-accessible galactose groups at glycan termini. If ECL lectin was used, F_L+IgG_ is specified as F_ECL+IgG_.

### Octagam contains a higher fraction of anti-OAβ IgG than Gammagard

**Fig 3:**
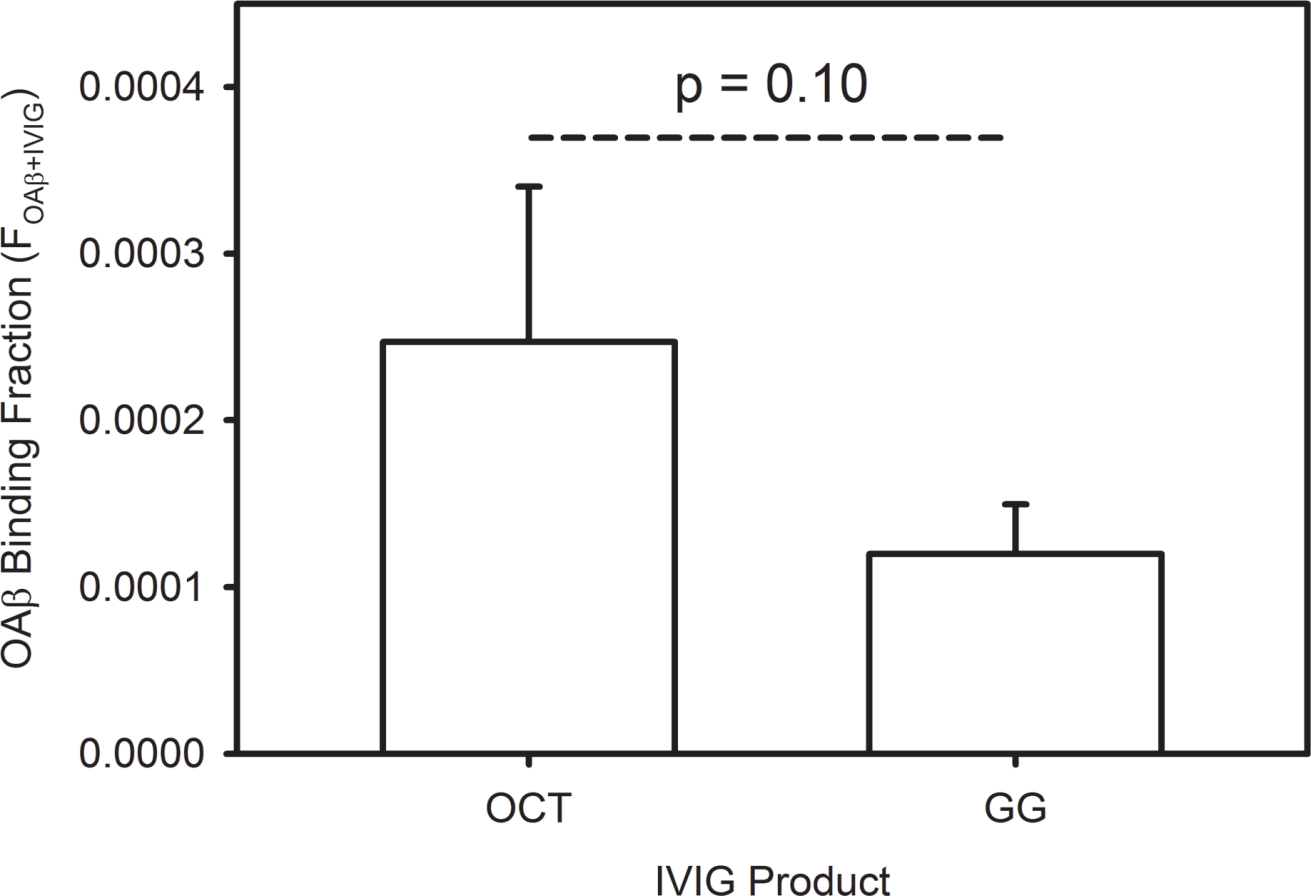
The fraction of anti-OAx IgG is higher in Octagam than in Gammagard. F_OAx+IVIG_ in IVIG products Octagam (OCT) and Gammagard (GG) was determined by CFCA. Error bars indicate standard deviations (n = 3) and the statistical significance of the difference between the two means is shown (p = 0.10).

CFCA was used to measure and compare F_OAβ+IVIG_ in Octagam and Gammagard (Fig 3). The result was that F_OAβ+IVIG_ for Octagam (0.00025) is approximately twice that of Gammagard (0.00012). The concentration of Octagam OAx^+^ IgG was reported at approximately 1.5 times that of Gammagard in a previous ELISA study [19]. As a possible explanation for the discrepancy in magnitude, Aβ peptides in the previous study had been disaggregated prior to immobilization. Nonetheless, both studies find that Octagam has a higher fraction of Ax-binding IgG than Gammagard.

The F_OAβ+IVIG_ determined here by CFCA for 10% Gammagard indicates an OAβ IgG concentration in this product that is approximately 2-fold higher (70 nM) than that measured in a previous ELISA study (~35 nM) [24]. While this difference is not excessive, the exact explanation is not known. One possible explanation is simply that slight differences in oligomer preparation and analytical methodology allow different or weaker-binding OAβ IgG binding in SPR versus ELISA. Another possible factor is that the nonspecific background binding from Gammagard was very high by ELISA, at ~50% of total OAβ^+^ IgG binding [24]. By contrast, in SPR measurements of IVIG, the background signal rise in the reference cell never exceeded 10% of that in the OAβ flow cell. Given the lack of nonspecific binding, CFCA may be providing a more accurate value of F_OAβ+IVIG_.

### Octagam and Gammagard have similar fractions of SNA-binding IgG

**Fig 4:**
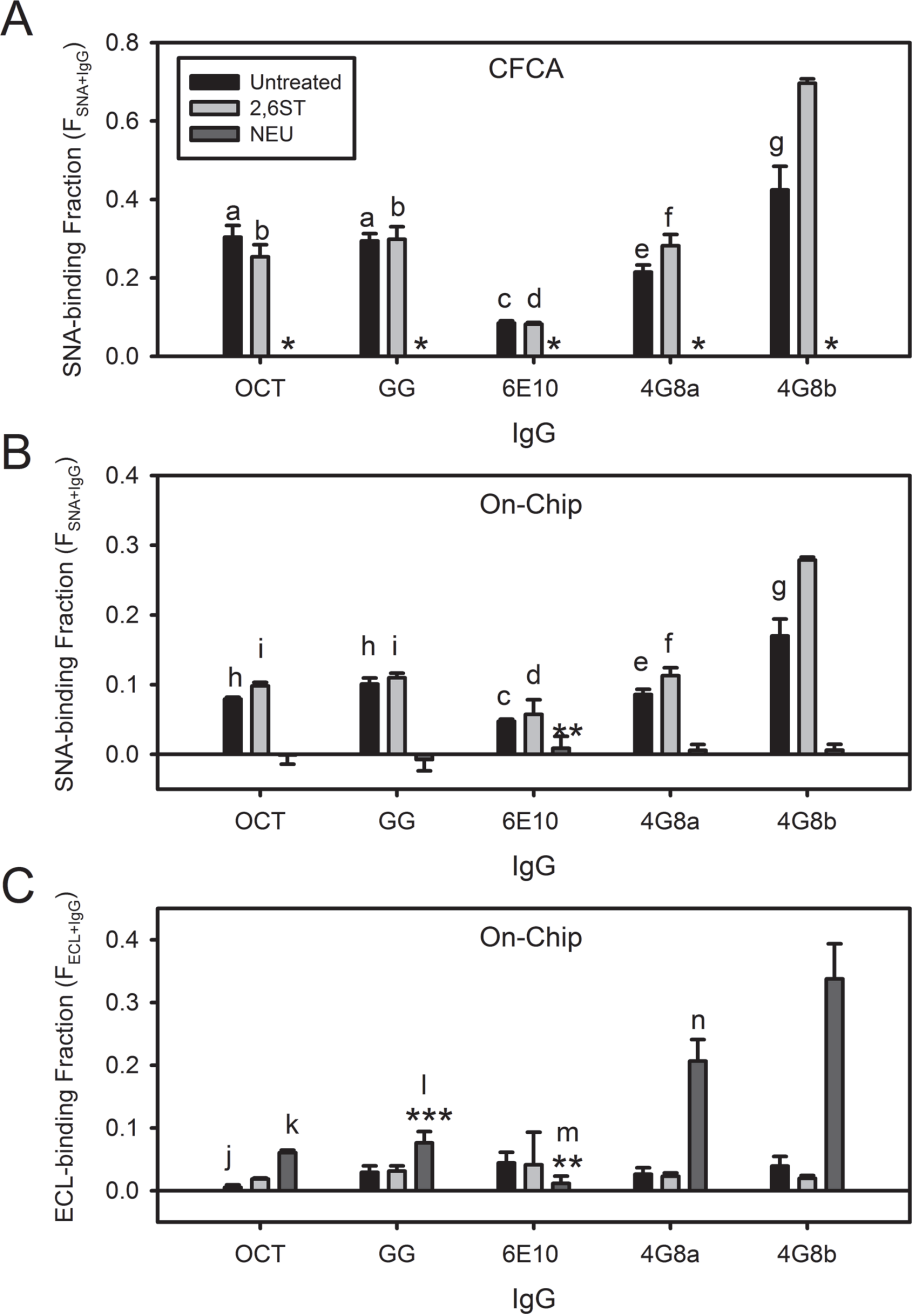
Surface sialic acid is similar between IVIG products and but varies in anti-Aβ monoclonal antibodies. Measurement of the SNA-binding fraction (F_SNA+IgG_) and ECL-binding fraction (F_ECL+IgG_) in Octagam IVIG (OCT), Gammagard IVIG (GG), mAb 6E10, and mAb 4G8 using CFCA (A) and the On-Chip method (B,C). Two different lots of mAb 4G8 are shown as 4G8a and 4G8b. Each IgG was enzymatically untreated (black bars), 2,6 sialyltransferase treated (2,6ST, light grey bars), and neuraminidase treated (NEU, dark grey bars). Error bars indicate standard deviations (n = 2). CFCA measurements of [IgG]_FC_SNA_ did not meet either the QC or SE criteria and F_SNA+IgG_ and are reported as zero (indicated with asterisk *). Statistical significant differences below p < 0.14 are shown for relevant comparisons, i.e. betweendifferent treatments of the same IgG and between different IgGs with the same enzymatic treatment condition. With the exception of On-Chip SNA/ECL analysis of 6E10 (**, 0.13 < p < 0.19) and On-Chip ECL analysis of GG (***, 0.11 < p <0.13), all NEU-treated IgG differed significantly (p < 0.08) from their corresponding untreated and 2,6ST-treated forms. Other relevant statistically significant differences are indicated as follows: (a) 0.05 < p < 0.10 between untreated 6E10 and 4G8a; (b) 0.02 < p < 0.08 between 2,6ST-treated 6E10 and 4G8b; (c) 0.04 < p < 0.08 between untreated 4G8a and 4G8b; (d) 0.05 < p < 0.11 between 2,6ST-treated 4G8a and p < 0.04 between 2,6ST-treated 4G8b; (e) 0.10 < p < 0.13 between 2,6ST-treated 4G8a and untreated 4G8b; (f) 0.01 < p < 0.03 between 2,6ST-treated 4G8b; (g) 0.08 < p < 0.10 between 2,6ST-treated 4G8b; (h) p < 0.05 between untreated 6E10 and 0.10 < p < 0.13 between untreated 4G8b; (i) p < 0.003 between 2,6ST-treated 4G8b; (j) p = 0.10 between 2,6ST-treated OCT; (k) 0.07 < p 0.11 between NEU-treated 6E10, 4G8a, and 4G8b; (l) 0.06 < p < 0.08 between NEU-treated 6E10, 4G8a, and 4G8b; (m) 0.06 < p 0.08 between NEU-treated 4G8a and 4G8b; (n) p = 0.13 between NEU-treated 4G8b. Differences with p > 0.14 are not shown.

Fig 4 shows a comparison of the fraction of SNA-binding and ECL-binding IgG in IVIG and of mAbs 6E10 and 4G8. In addition, the values of F_SNA+IgG_ and F_ECL+IgG_ are determined in untreated IgG products after treatment with 2,6 sialyltransferase (2,6ST) and after treatment with neuraminidase (NEU). Fig 4A and 4B show values of F_SNA+IgG_, determined by CFCA and the On-Chip method respectively. Fig 4C confirms these findings with values of F_ECL+IgG_ determined by the On-Chip method. Due to a high variability noted for F_SNA+IgG_ and F_ECL+IgG_ between lots of mAb 4G8, the individual 4G8 lot values are shown as 4G8a and 4G8b.

Both Fig 4A and 4B show a similar profile of F_SNA+IgG_ values. Untreated 4G8b is marginally higher than untreated 4G8a, Octagam, and Gammagard, with all four untreated IgGs being higher than untreated 6E10. Treatment with 2,6ST produced no increase in the IgG except a statistically marginal increase in 4G8a (0.10 < p < 0.13) and 4G8b (0.08 < p < 0.10). By contrast, NEU-treatment produced a statistically significant reduction in all IgG to F_SNA+IgG_ ~ 0. The exception is On-Chip 6E10 in Fig 4B, which showed marginal statistical significance (0.10 < p < 0.13) between untreated and NEU-treatment conditions. This is due largely to similar mean values of 6E10 F_SNA+IgG_ that are close to zero and is not the result of high error in measurements of this IgG species.

Fig 4C is consistent with Fig 4A and 4B because F_ECL+IgG_ is expected to be inversely correlated with F_SNA+IgG_. Sialylated glycan termini do not bind ECL and removal of sialic acid will yield a terminal galactose with affinity for ECL. A notable exception is that a higher F_ECL+IgG_ is found for untreated and 2,6ST-treated 6E10 versus NEU-treated 6E10. Although this difference is of weak statistical significance (0.13 < p < 0.19), it is clearly not the inverse of the 6E10 F_SNA+IgG_ values shown in Fig 4B. Excepting this 6E10 case, the F_ECL+IgG_ of all other NEU-treated IgG were higher than their corresponding untreated and 2,6ST-treated forms. This NEU-treated increase in F_ECL+IgG_ was statistically significant (p < 0.08) for Octagam, 4G8a, and 4G8b and marginally significant for Gammagard (0.11 < p < 0.13). In addition, a small but statistically significant increase in F_ECL+IgG_ was found when untreated Octagam was treated with 2,6ST. Despite these minor exceptions, the magnitude of F_ECL+IgG_ of a given IgG sample in Fig 4C is generally inversely correlated with the values of F_SNA+IgG_ in Fig 4A and 4B.

Despite similar trends in Fig 4A and 4B, a notable difference was that CFCA consistently predicted a higher value of F_SNA+IgG_ that the On-Chip method. With no biochemical standard of F_SNA+IgG_ available, it was unclear whether F_SNA+IgG_ is more accurately determined from CFCA or the On-Chip method. Fractional yields of IVIG from SNA affinity chromatography have been reported between 0.10–0.13 [29,39]. These literature values fall closer to the On-Chip measurement of F_SNA+IgG_ (0.08–0.10) than those by CFCA (~0.30). However, SPR sensor binding measurements and column purification yields involve different factors. This issue required empirical investigation to resolve.

A logical supposition is that CFCA is more accurate because IgG bound in the On-Chip method would not expose all available SNA binding sites. To test this hypothesis, a control experiment was needed in which an analyte with known binding stoichiometry to IgG could be substituted for SNA in Step 2. This control experiment was possible if 4G8 was bound to immobilized OAβ in Step 1 and analyte SpA used to bind this IgG2 mouse subtype in Step 2. Previous studies have shown empirically that 1:1 Fc:SpA binding predominates at excess SpA [53,56]. Given the SpA mass of 42 kD and IgG mass of 150 kD, a Step2/Step1 signal ratio of 0.280 is expected if all binding sites available in solution remain available after 4G8 is immobilized.

**Fig 5:**
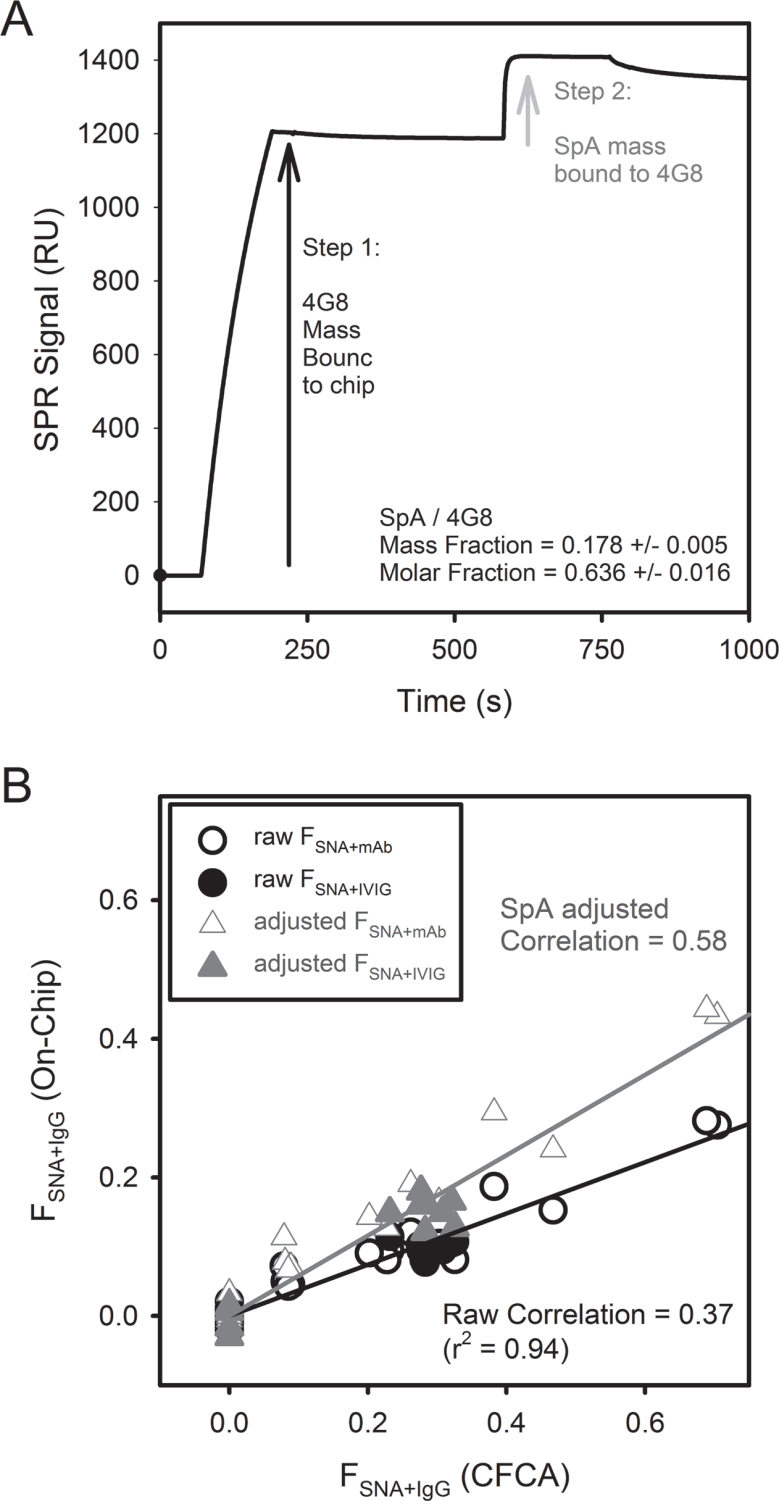
Steric hindrance at IgG binding sites increases after IgG binds ligands on the sensor surface. (A) Representative SPR On-Chip experiment on a 60-fold dilution of 4G8 captured in a flow cell with immobilized OAβ. In Step 1, IgG (4G8) is bound for 120 s and the association binding signal RU(4G8) measured. In Step 2, 1 μM SpA is bound for 180 s to the flow cell containing 4G8 and this second association signal RU(ProteinA+4G8) is measured. Background binding signal RU(ProteinA-4G8) of 1 μM SpA was very low (6–8 RU) in the absence of 4G8 (not shown). Also shown are the mass fraction corresponding to RU(ProteinA+4G8) / RU(4G8) and the molar fraction which is the mass fraction multiplied by the mass ratio of 4G8/SpA = 3.57. (B) Correlation between F_SNA+IgG_ determined by CFCA (x-axis) and F_SNA+IgG_ determined by the On-Chip method (y-axis). Each data point is the cross-correlation point for an untreated, 2,6ST-treated, or NEU-treated IgG data point from CFCA in Fig 4A with the same sample from On-Chip analysis in Fig 4B (black circles) or after adjustment with a correction factor determined from SpA molar fraction (1.57) (grey triangles). CFCA values where SNA quantitation did not meet the SE/QC criteria were given a value of F_SNA+IgG_ = 0. For comparison, data points for IVIG (closed symbols) and mAb (open symbols) are distinguished. The linear fit, slope and r^2^ for the raw On-Chip correlation (black line) and SpA-adjusted On-Chip correlation (grey line) with CFCA values are shown.

Fig 5A shows a representative SPR sensogram of this control measurement, with a Step 2/Step 1 RU ratio of 0.178 +/- 0.005. This value is less than the expected value of 0.28 and corresponds to an On-Chip molar ratio of SpA:4G8 of 0.636. This result supports the premise that IgG binding to the sensor ligand results in an increase in steric hindrance for subsequently introduced IgG-binding analytes. Having established that SpA binding sites were 36.4% less accessible after IgG binding to the sensor chip, it was logical to ask if this same level of steric hindrance was also found with SNA binding sites.

To address this question, the On-Chip values of F_SNA+IgG_ in Fig 4B were multiplied by the reciprocal of the SpA:4G8 molar ratio, 1/0.636 = 1.57, and the results are shown in Fig 5B. In Fig 5B, the correlation of these SpA-adjusted On-Chip values versus CFCA (grey data points) is shown along with raw On-Chip values versus CFCA (black data points). The raw correlation between On-Chip values and CFCA in Fig 5B did not differ significantly between mAb data (black open circles, 0.38) and IVIG data (black filled circles, 0.34). Steric hindrance at SNA-binding sites in the On-Chip method was deemed to be similar regardless of whether mAb was bound to the OAβ chip or IVIG was bound to the Protein A chip. Using mAb and IVIG data together, Fig 5B shows that the total On-Chip versus CFCA slope increases from 0.37 (black points/line) to 0.58 (grey points/line) after the SpA-adjustment is applied.

The empirical 1.57 SpA adjustment factor corrects for some, but not all, of the 2.7-fold discrepancy in F_SNA+IgG_ between CFCA and the non-adjusted On-Chip data. Because the adjusted slope remains less than 1.0 after SpA-adjustment, the steric hindrance of SpA appears to be less than that of SNA. It is concluded that the degree of steric hindrance for surface-bound IgG cannot be assumed for different analytes with different molecular configurations and binding properties. For example, SpA is smaller than SNA and likely encounters less steric repulsion when binding. In addition, an analyte with polyvalent capability would more effectively hinder its fellow analytes from binding IgG binding by occupying multiple sites. While a full investigation of such factors is outside the scope of the present study, the relevant finding in Fig 5 is that analyte binding to surface-bound IgG is sterically hindered. CFCA is therefore presumed to be more accurate for F_SNA+IgG_ determination.

While the On-Chip method may not be accurate as CFCA for F_SNA+IgG_ determination, it did demonstrate high precision. This high precision is shown in Fig 5B by the high correlation coefficient of the linear fit between CFCA and the On-Chip data (r^2^ = 0.94). Also, the overall slope was similar between IVIG data (F_SNA+IVIG_, filled symbols) and mAb data (F_SNA+mAb_, open symbols). Thus, the On-Chip method can be used to precisely compare relative changes in F_SNA+IgG_ between different IgG preparations and with different sensor chips. Because CFCA was used as a benchmark in the present study, an empirical correction factor of 2.7 for all On-Chip values of F_SNA+IgG_ can be used to adjust the On-Chip values to be consistent with CFCA. This correction factor of 2.7 only applies to SNA and not to other analytes used in Step 2 of the On-Chip analysis.

### IVIG surface-accessible sialic acid is predominantly located on the Fab domain

**Fig 6:**
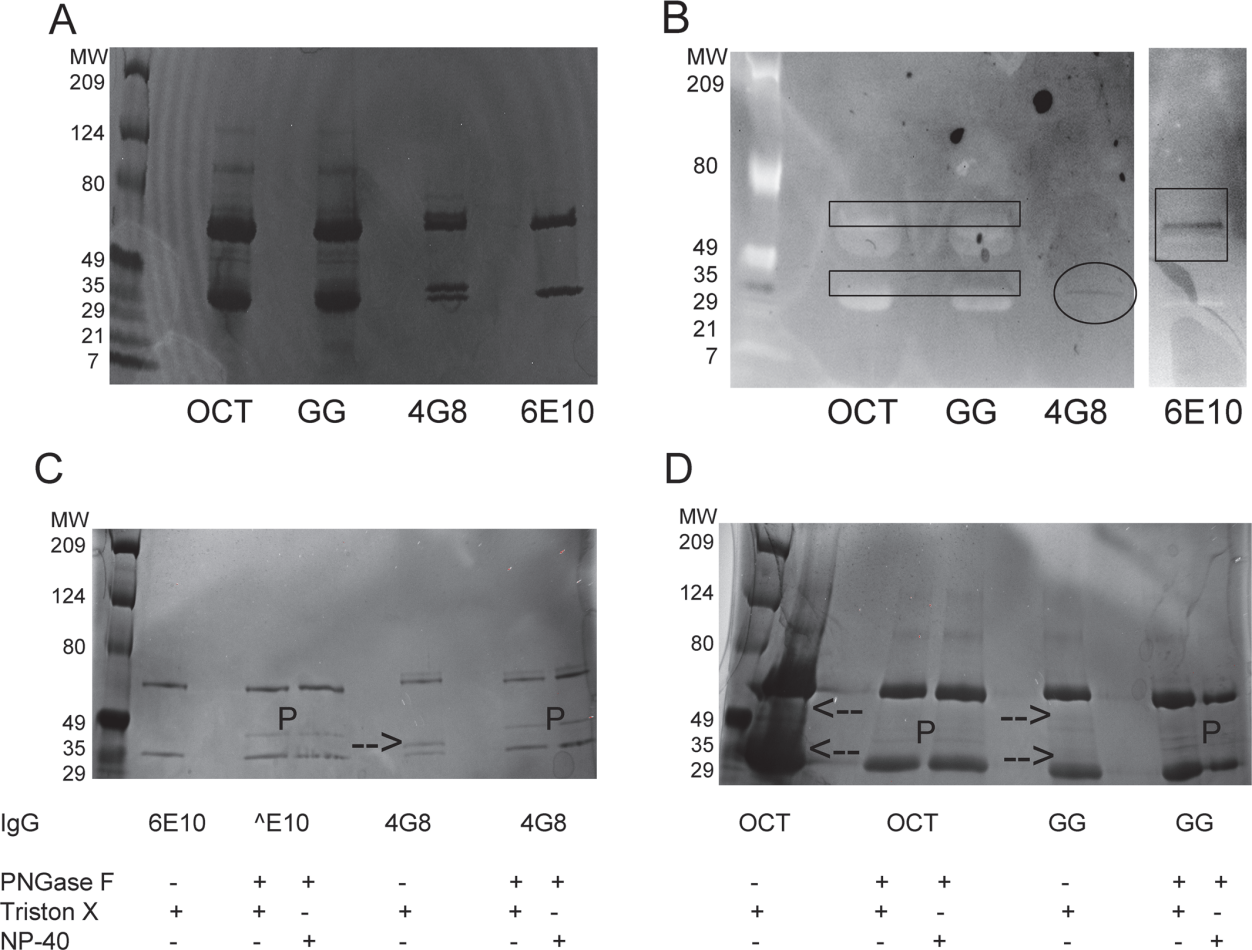
4G8 is IgL sialylated, 6E10 is Fc sialylated, and IVIG exhibits both Fc and IgL sialylation. (A) SDS-PAGE gel of Octagam IVIG (OCT), Gammagard IVIG (GG), 4G8, and 6E10. (B) SNA lectin blot of Octagam IVIG (OCT), Gammagard IVIG (GG), 4G8, and 6E10. Diffuse dark staining in the SNA lectin blot is evident immediately above both light chain (IgL) and heavy chain (IgH) bands of IVIG (black rectangles), in the upper of two IgL bands of 4G8 (circle), and in the single IgH band of 6E10 (square). (C,D) SDS-PAGE gel of 6E10 and 4G8 (C) and OCT and GG (D), all treated with and without PNGase F. PNGase F-treated samples contained one of two surfactants, 10% Triton X-100 or 15% NEB. For comparison, samples without PNGase F contained 10% Triton X-100. Bands migrating as indicated by “P” are the PNGase F enzyme (36 kD). Arrows in non-PNGase F lanes indicate IgG bands that are absent after PNGase F treatment.

To determine if Octagam and Gammagard differ in sialic acid location, SDS-PAGE and lectin blots were used initially (Fig 6). SDS-PAGE in Fig 6A shows that Octagam and Gammagard are comprised of light (IgG_L_) and heavy (IgG_H_) chain bands near the expected masses of 25 and 50 kD respectively. However, a clear picture of IgG_L_ and IgG_H_ states in IVIG is complicated by diffuse primary IgG_L_ and IgG_H_ bands and also many higher molecular weight bands. The bands of mAbs 4G8 and 6E10 were much simpler than those of IVIG. While simpler than IVIG, 4G8 does exhibit two well-resolved bands for both IgG_L_ and IgG_H_. By contrast, 6E10 shows only a single discernible band for both IgG_L_ and IgG_H_. One explanation for additional IgG_L_ and IgG_H_ bands in Fig 6A is the presence of glycans bound in the respective V_L_ and V_H_ regions of these chains [29,39].

SNA lectin blotting was used to assess the degree of sialylation present in these IgG bands (Fig 6B). When imaging high protein concentrations using WesternDot on a Dark Reader, both non-fluorescent proteins are observed (light bands) along with the blot-specific bands (dark bands). For IVIG bands in Fig 6B, one notes a weak intensity of blotting on the upper portion of both the heavy and light chain bands (rectangles). SNA blotting of 4G8 is most pronounced on the heavier of the two IgG_L_ bands (circle), indicating that 4G8 sialylation occurs primarily on Fab glycans. SNA blotting is found only on the IgG_H_ band of 6E10 (square).

To further assess whether the slower migrating bands are the result of additional N-linked glycans to IgG chains, mAb samples (Fig 6C) and IVIG samples (Fig 6D) were run untreated and after treatment with PNGase F. In Fig 6C, 6E10 shows no discernable difference in bands excepting the added PNGase F band indicated by “P” at 36 kD. By contrast, the heavier IgG_L_ band of 4G8 (highlighted by arrow), but not the heavier IgG_H_ band, is removed after PNGase F treatment. PNGase F treatment of IVIG is complicated by the presence of multiple bands (Fig 6D). Despite this complexity, two band regions of untreated IVIG are noted with reduced intensity after PNGase F treatment. Similar to 4G8, one such band migrates slightly slower than IgG_L_ (lower arrow). In addition, an upper band that migrates slightly faster than IgGH is not evident in the PNGase F lanes (upper arrow). Taken together, the primary sialylation sites appear to be limited to the conserved Fc glycan (6E10, IVIG) or to a lone additional glycan bound to the IgG_L_ domain (4G8, IVIG). Although 4G8 and IVIG bands are observed that migrate slower than IgG_H_, their insensitivity to PNGase F treatment indicates they do not possess N-linked glycans.

In Fig 6, sialylation on 6E10 appears entirely on the Fc domain while sialylation on 4G8 appears entirely on the Fab domain. Thus, SNA binding to these mAbs is expected to occur with sialic acid occupying one of these two domains exclusively. For IVIG, the relative fraction of total sialic acid on Fc and Fab domains is more difficult to quantify in Fig 6. In addition, it has been reported that Fab sialic acid preferentially binds SNA over Fc sialic acid. To resolve this ambiguity, the primary domain of surface sialic acid in Octagam and Gammagard was determined through enzymatic treatment with Fc-specific endoglycosidase EndoS.

Given the quantitative limitations of the Lectin Blots, SPR was used to quantitatively determine the fraction of accessible sialic acid associated with the conserved Fc glycan. For this experiment, IgG was treated with and without EndoS enzyme which selectively cleaves Fc glycans. The remaining fraction of SNA-binding attributed to non-Fc glycans (F_EndoS_) was then determined using Eq. 6 [57]. A lower value of F_EndoS_ corresponds to a greater fraction of surface sialic acid associated with the Fc glycan. A high value of F_EndoS_ indicates that most surface sialic acid is associated with other glycans in the Fab domain.

**Fig 7:**
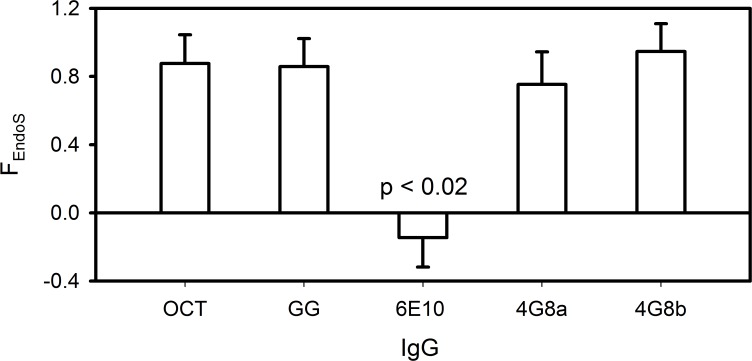
Surface sialic acid predominantly resides in the Fab domains of 4G8 and IVIG and in the Fc domain of 6E10. The fraction of untreated SNA-binding IgG remaining after removal of Fc glycans with EndoS treatment (F_EndoS_) of Octagam IVIG (OCT, n = 3), Gammagard IVIG (GG, n = 2), 6E10 (n = 2), 4G8a (n = 2), and 4G8b (n = 3). Error bars represent standard deviations. A statistically significant difference is found between 6E10 and all other IgG (p < 0.02) and all other IgG means differed from each other with p > 0.20.

Fig 7 shows results for 6E10 and 4G8 that are consistent with Fig 6. Briefly, F_EndoS_ is close to zero for 6E10 and close to 1.0 for 4G8a and 4G8b. For Octagam and Gammagard, F_EndoS_ was shown to be similar to 4G8 at approximately 0.9. Consistent with previous reports, surface sialic acid is primarily associated with Fab glycans in most IVIG antibodies.

### OAβ^+^ IgG in IVIG contains surface sialic acid lacking the α2,6 linkage

Significant levels of OAβ^+^ IgG (Fig 3) and surface sialic acid (Fig 4) were detected in Octagam and Gammagard IVIG. Because these two individual properties are potentially relevant to AD treatment, IgG subgroups with both of these properties in IVIG are of particular interest. To date, this combined measurement has not been performed in IVIG. CFCA is accurate but only can only measure one of these two properties at a time. Having been validated in the present study, the On-Chip method was appropriate for quantifying the IgG sub-subpopulation in IVIG with both OAβ^+^ and SNA^+^ or ECL^+^ binding affinities. Analogous to the 4G8 experiment shown in Fig 2, OAβ^+^ IgG from IVIG was captured in Step 1 and excess SNA or ECL lectin binding was performed on the captured OAβ^+^ IgG in Step 2.

**Fig 8:**
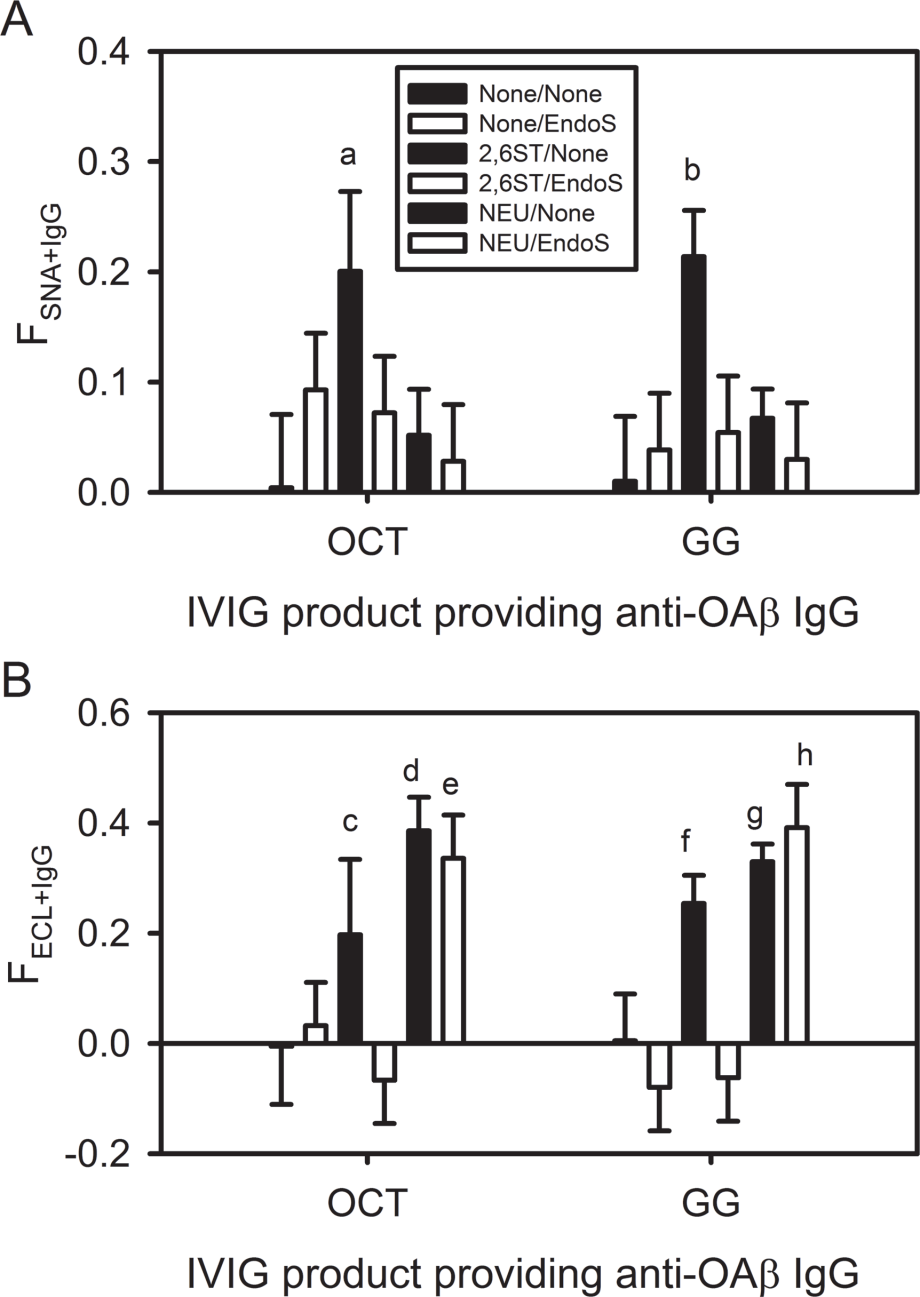
OAβ^+^ IgG in IVIG exhibits a high fraction of Fab surface sialic acid lacking α2,6 linkages. On-Chip SPR measurement of (A) the SNA-binding fraction (F_SNA+IgG_) and (B) the ECL-binding fraction (F_SNA+IgG_) of OAβ^+^ IgG in Octagam IVIG (OCT) and Gammagard IVIG (GG). In each set of six, bars 1,2 indicate results without 2,6ST or NEU pre-treatment, bars 3,4 indicate results with 2,6ST pre-treatment, and bars 5,6 indicate NEU pre-treatment. Back bars indicate no post-treatment and white bars indicate EndoS post treatment. Error bars represent standard deviations (n = 2). The statistical significance of the difference in the relevant means for comparison (same lectin analysis, same IVIG, same pre- or post- treatment condition) is indicated as follows: (a) 0.10 < p < 0.20 between None/None, 2,6ST/EndoS, and NEU/None OCT by SNA; (b) 0.05 < p < 0.08 between None/None, 2,6ST/EndoS, and NEU/None GG by SNA; (c) p = 0.17 between 2,6ST/EndoS OCT by ECL; (d) p = 0.07 between None/None OCT by ECL; (e) 0.03 < p < 0.06 between None/EndoS and 2,6ST/EndoS OCT by ECL; (f) 0.05 < p < 0.09 between None/None and 2,6ST/EndoS GG by ECL; (g) p = 0.08 between None/None GG by ECL; (h) p = 0.03 between None/EndoS and 2,6ST/EndoS GG by ECL. Differences with p > 0.20 are not shown.

Fig 8A and 8B show the values of F_SNA+IgG_ and F_ECL+IgG_ respectively of OAβ^+^ IgG in untreated, 2,6ST-treated, and NEU-treated IVIG. In addition, each enzymatic treatment of IVIG was performed with and without EndoS post-treatment to determine the extent of Fc glycan contribution to F_SNA+IgG_ and F_ECL+IgG_. Fig 8A shows that only 2,6ST-treated OAβ^+^ IgG shows a higher value of F_SNA+IgG_ ~ 0.2 versus the other treatment conditions, which exhibited statistically insignificant values. While the statistical significance of this measurement is marginal because of a low OAβ^+^ IgG binding response on the chip, a value of 2,6ST-treated F_SNA+IgG_ of ~ 0.2 was observed for both Octagam (0.10 < p < 0.20) and Gammagard (0.05 < p < 0.08). The lower value of F_SNA+IgG_ for 2,6ST/EndoS-treated OAβ^+^ IgG indicates that the substrate for this enzymatic sialylation is the Fc glycan.

Compared with Fig 8A and Fig 8B shows surprising results from F_ECL+IgG_ measurements. Untreated IVIG did not show a significant F_ECL+IgG_. However, 2,6ST treatment produced an unexpected high value of F_ECL+IgG_ ~ 0.2. Although statistically marginal, this increased value was found for both Octagam (p = 0.17) and Gammagard (0.05 < p < 0.09) and was not observed after 2,6ST/EndoS treatment for both IVIG. A second unexpected result in Fig 8B was the finding that both NEU-treatment and NEU/EndoS-treatment produced a high value of F_ECL+IgG_ ~ 0.35 for OAβ^+^ IgG. This was observed in both Octagam and Gammagard. Thus, endogenous human OAβ^+^ IgG exhibits surface sialic acid that does not bind SNA and is likely of a different biochemical linkage than that of 6E10, 4G8, and most other human IgG antibodies.

## Discussion

The results demonstrate that the properties of Octagam and Gammagard studied here are quite similar. The largest difference was F_OAβ+IVIG_ in Octagam being approximately twice that of Gammagard, though it was of marginal statistical significance (p = 0.10 in Fig 3). This result is consistent with a previous ELISA study and is a second example showing Octagam with more Aβ-specific IgG than Gammagard [19]. Both results shed light on the clinical hypothesis that AD patients would benefit from increased levels of anti-Aβ IgG [10,18].

Assuming a 400 mg/kg IVIG dose, 100 kg patient, 7 liters of blood, 100 ml of cerebrospinal fluid (CSF) and 0.1% total IgG peak brain penetration, picomolar OAβ^+^ IgG concentrations in the CSF are expected in the peak timeframe after IVIG infusion [58]. This estimate is supported by a small study of patient CSF after Octagam treatment which indicated an increase of anti-Aβ IgG CSF concentration from 10 to 70 pM [10]. Because estimates of OAβ^+^ IgG binding thermodynamics estimate a nanomolar affinity, variation of picomolar CSF OAβ^+^ IgG concentrations could possibly result in different clincial outcomes [18,27,36]. Given the elevated F_OAβ+IVIG_ in Octagam versus Gammagard, this clinical hypothesis predicts that Octagam would show more positive clinical outcomes than Gammagard. Recent clinical trials reported small and patient-selective benefits from Gammagard and no benefit from Octagam treatment [2,3]. Thus, the clinical relevance of natural anti-Aβ IgG alone remains unclear.

OAβ^+^ IgG and surface sialic acid did not correlate with the reported clinical outcomes of Octagam and Gammagard. However, unique glycosylation properties of the OAβ^+^ IgG subgroup in IVIG were identified. Modulation of these unique glycosylation features might lead to better IVIG treatments for AD. The primary finding here was that approximately one third of OAβ^+^ IgG in IVIG exhibited terminally-sialylated Fab glycans completely lacking the standard α2,6 linkage to galactose (Fig 8).

Examination of Fig 8A shows F_SNA+IgG_ ~ 0 for both untreated and NEU-treated OAβ^+^ IgG. Surface sialic acid with the α2,6 glycan linkage is therefore not present in endogenous OAβ^+^ IgG. In Fig 8B, untreated OAβ^+^ IgG also showed a F_ECL+IgG_ ~ 0, indicating a lack of any surface glycans with terminal galactose in endogenous OAβ^+^ IgG. However, the high value of F_ECL+IgG_ ~ 0.35 in NEU-treated OAβ^+^ IgG unveiled a significant number of galactose groups after an apparent cleavage of many SNA-insensitive sialic acids. To account for the lack of SNA binding in untreated samples, the naturally occurring sialic acid in endogenous OAβ^+^ IgG must be bonded to galactose through linkages other than the α2–6 orientation. The similar F_ECL+IgG_ values between NEU-treated and NEU/EndoS-treated OAβ^+^ IgG further indicate that the non-α2,6 sialic acid groups reside in the Fab domain.

Another notable finding in the OAβ^+^ IgG glycosylation results in Fig 8A and 8B is that 2,6ST treatment yielded OAβ^+^ IgG with increased binding affinity to both SNA and ECL. Because 2,6ST/EndoS treatment eliminated both SNA and ECL binding, it is concluded that both lectins are binding at the Fc glycan of 2,6ST-treated OAβ^+^ IgG. The ECL sensitivity to EndoS of OAβ^+^ IgG differs from that of 4G8 and IVIG but not 6E10. Like OAβ^+^ IgG, 6E10 also showed a higher FECL+IgG after 2,6ST treatment in Fig 4C and highlights two possibilities. First, 2,6ST-treatment may not completely sialylate the sterically hindered Fc glycans. Second, this partial Fc sialylation may alter the IgG structure such that residual Fc galactose groups are more exposed. The different magnitudes of F_ECL+IgG_ after 2,6ST-treatment of human polyclonal OAβ^+^ IgG in Fig 8B (F_ECL+IgG_ ~ 0.2) and mouse monoclonal 6E10 in Fig 4C (F_ECL+IgG_ ~ 0.04) are not fully explained at present and require further study.

A previous LC/MS study reported N297 Fc glycan sialylation to be similar between IVIG and its anti-Aβ IgG subgroup [41]. However, the present study and others shows that surface sialic acid in the Fab domain is primarily responsible for SNA binding in untreated IVIG, with minimal contribution from sialic acid on the Fc glycan [29,39,40]. Because the present study does not rule out the presence of buried sialic acid at the N297 glycan of OAβ IgG, the results here are not in conflict with this prior LC/MS study [41].

The absence of α2,6-linked surface sialic acid in natural OAβ^+^ IgG reported here is consistent the previous study with a mouse model of nephrotoxic nephritis [28]. In this study, antigen-specific IgG showed significantly lower levels of SNA binding after a secondary exposure to the antigen [28]. While a valuable study, the authors suggested that the loss of SNA binding was due to the loss of sialic acid and did not investigate if sialic acid was actually present on the IgG in non-α2,6 linkages. The present results demonstrate that non-α2,6 linked sialic acid can also occur in antigenic IgG.

It remains unclear whether an anti-OAβ immune response helps AD patients or if it instead leads to unintentional exacerbation of AD pathology [17]. The present study provides perspective on this question because it demonstrates that OAβ^+^ IgG in Octagam and Gammagard clinical trials was delivered to AD patients with sialic acid in an atypical biochemical linkage to galactose (Fig 8). It is not known if current anti-Aβ antibody drugs for AD also present atypical sialic acid linkages on their surface [4,5]. A mass spectroscopy analysis of N-glycans cleaved from multiple commercial therapeutic antibodies indicated that the sialic acid level in these antibody drugs is typically very low [59]. As an exception, glycans of HIV-neutralizing antibody 4E10 are found to be highly sialylated and can preferentially exhibit α2,3 or α2,6 linkages depending on expression conditions [59]. Extracellular expression of α2,3 and α2,6 sialic acid levels on membrane glycoproteins delineates species susceptibility to viral infection [60]. However, the biological impact of different sialic acid linkage isomers is less clear when part of proteins such as IgG [60].

One new route of investigation for AD treatment would be to investigate natural human anti-Aβ oligomer antibodies with glycans presenting the typical α2,6-linked surface sialic acid moiety. The α2,6-linked sialic acid can be produced using initial NEU treatment, followed by2,6ST treatment and/or enrichment with SNA affinity chromatography [29,39,40,61]. This newer formulation of α2,6-sialylated OAβ^+^ IgG may more effectively reduce neuroinflammation in addition to reducing OAβ levels in the brain [16,29,61]. Evidence also suggests that surface sialic acid properties modulate protein permeability through the blood-brain barrier, a property that could enhance IgG treatment of AD [62,63]. Given the potential of antibody therapy for AD, continued efforts to modulate bioactivity of existing therapeutic IgG through changes in surface glycosylation are warranted.

Another relevant finding is that endogenous IVIG surface sialic acid is primarily located on Fab glycans. A debate exists in the literature regarding the primary location of surface sialic acid in IVIG. This question is relevant to IVIG clinical applications because enrichment of sialylated IgG after affinity chromatography with SNA lectin is associated with anti-inflammatory outcomes in select autoimmune disease models [28,29,61]. A number of studies have inferred the location of this “functional” sialic acid in IVIG by measuring the change in Fab and Fc sialylated glycan populations before and after SNA affinity chromatography [29,39,40]. The work here also addressed the question of surface sialic acid location in IVIG but with different experimental tools, i.e. SPR and enzymatic treatments. All studies in the present work reach the same conclusion—that IVIG surface sialic acid with SNA affinity is predominantly associated with N-linked Fab glycans. Additionally, Fab surface sialic acid also predominates on the OAβ^+^ IgG subgroup but with a linkage orientation that prevents SNA binding.

SNA binding of IVIG and 4G8 antibodies is primarily mediated by sialylated glycans in the Fab domains. However, the present results with 6E10 (Fig 4A and 4B) and 2,6ST-treated OAβ^+^ IgG (Fig 8A) do show that sialylated Fc glycans can also facilitate SNA binding. Binding between sialylated Fc glycans and SNA on affinity columns has been noted previously for IgG preparations where sialylated Fab glycans were not present [29,39]. Thus, sialylated Fc glycans are not completely inaccessible on the IgG surface to lectin binding.

Previous studies also indicate that Fc sialic acids are more sterically hindered and do not bind SNA as well as Fab glycan sialic acids [39]. This idea was supported by lower sialic acid enrichment efficiency of isolated Fc domains relative to Fab fragments using SNA affinity chromatography [29]. The low F_SNA+IgG_ ~0.04 of 2,6ST-treated 6E10 in Fig 4A and 4B is consistent with these previous studies. However, the relatively high F_SNA+IgG_ ~0.20 of 2,6ST-treated OAβ^+^ IgG in Fig 8A results from Fc sialylation and exceeds that of Fab-sialylated total IVIG (F_SNA+IgG_ ~0.10) in Fig 4B. Thus, factors other than Fc/Fab domain location may influence the surface-accessibility of terminal sialic acids on IgG glycans.

The biological activity of Fc and Fab glycan sialic acid moieties, and their different possible biochemical orientations, is largely unknown. Enrichment of IgG from IVIG with affinity for SNA has been shown to reduce inflammation in some disease models [28,29]. The exact mechanism of how this occurs is not known although altered antigen binding may be involved [29]. While Fc sialic acid is minimally enriched by SNA affinity chromatography of IVIG, it is also possible that Fc sialylation may alter IgG biological activity. One proposed hypothesis is that sialylation of the Fc glycan changes the Fc structure through internal interactions which alters affinities between different binding partners [64]. Testing this hypothesis is beyond the scope of the present study but this is an intriguing subject for future work.

From a methodological perspective, the present work demonstrates the use of two complementary SPR methods for the study of subgroups in complex polyclonal IgG preparations. CFCA is the best method to obtain accurate concentration measurements of IgG with a single binding property. The On-Chip method proved to be useful in measuring an IgG sub-subfraction within polyclonal IgG preparations capable of binding two separate proteins. Using these two methods together is important because future antibody research for AD will likely develop antibodies that bind multiple targets [65]. Efforts are currently underway to develop bifunctional antibodies that both bind the blood-brain barrier and also target Aβ peptides [66]. In line with these efforts, the two SPR methods used here provide useful tools to accurately characterize the next generation of antibody therapeutics for AD.

## Supporting Information

S1 Table Tables of data and errors shown in Figs. 3, 4A, 4B, 4C, 5B, 7, 8A and 8B. IgG comprising x-axis categories in Figs. 3, 4A, 4B, 4C, and 7 are shown in the far left column. The IgG x-axis categories in Fig 8A are shown under the column titled “SNA” and the IgG x-axis categories in Fig 8B are shown under the column titled “ECL”. Means and standard deviations shown on the y-axis of Figs. 3, 4A, 4B, 4C, 7, 8A and 8B are listed under column titles “Mean” and “SD” respectively. For Fig 4A and 4B data, y-axis means and standard deviations of untreated, 2,6-sialyltransferase-treated, neuraminidase-treated IgG are designated with the column titles “Untreated”, “2,6ST”, and “NEU” respectively. For Fig 5B, the raw x-axis are shown under the column “CFCA”, the unadjusted y-axis On-Chip raw data are shown under the column “On-Chip (raw)”, and the adjusted y-axis On-Chip raw data are shown under the column “On-Chip (adjusted)”. For Fig 5B data, the x-y pairs of all IgG data, IVIG data alone, and monoclonal antibody data alone are designated with the column titles “All data”, “IVIG data”, and “mAb data” respectively.
(PDF)
