# Supplementary material for: Anti-Aβ Oligomer IgG and Surface Sialic Acid in Intravenous Immunoglobulin: Measurement and Correlation with Clinical Outcomes in Alzheimer’s Disease Treatment
Source: PLoS One. 2015 Mar 31;10(3):e0120420. doi: 10.1371/journal.pone.0120420 (PMC4380445; doi:10.1371/journal.pone.0120420)
Supplement: S1 Table — IgG comprising x-axis categories in Figs. 3, 4A, 4B, 4C, and 7 are shown in the far left column. The IgG x-axis categories in Fig 8A are shown under the column titled “SNA” and the IgG x-axis categories in Fig 8B are shown under the column titled “ECL”. Means and standard deviations shown on the y-axis of Figs. 3, 4A, 4B, 4C, 7, 8A and 8B are listed under column titles “Mean” and “SD” respectively. For Fig 4A and 4B data, y-axis means and standard deviations of untreated, 2,6-sialyltransferase-treated, neuraminidase-treated IgG are designated with the column titles “Untreated”, “2,6ST”, and “NEU” respectively. For Fig 5B, the raw x-axis are shown under the column “CFCA”, the unadjusted y-axis On-Chip raw data are shown under the column “On-Chip (raw)”, and the adjusted y-axis On-Chip raw data are shown under the column “On-Chip (adjusted)”. For Fig 5B data, the x-y pairs of all IgG data, IVIG data alone, and monoclonal antibody data alone are designated with the column titles “All data”, “IVIG data”, and “mAb data” respectively. (PDF) [file pone.0120420.s001.pdf]

S1 Table. Data and errors shown in Figures 3, 4A, 4B, 4C, 5B, 7, 8A, and 8B. IgG comprising x-axis categories in Figures 3, 4A, 4B, 4C, and 7 are shown in the far left column. The IgG x-axis categories in Figure 8A are shown under the column titled "SNA" and the IgG x-axis categories in Figure 8B are shown under the column titled "ECL". Means and standard deviations shown on the y-axis of Figures 3, 4A, 4B, 4C, 7, 8A, and 8B are listed under column titles "Mean" and "SD" respectively. For Figure 4A and 4B data, y-axis means and standard deviations of untreated, 2,6-sialyltransferase-treated, neuraminidase-treated IgG are designated with the column titles "Untreated", "2,6ST", and "NEU" respectively. For Figure 5B, the raw x-axis are shown under the column "CFCA", the unadjusted y-axis On-Chip raw data are shown under the column "On-Chip (raw)", and the adjusted y-axis On-Chip raw data are shown under the column "On-Chip (adjusted)". For Figure 5B data, the x-y pairs of all IgG data, IVIG data alone, and monoclonal antibody data alone are designated with the column titles "All data", "IVIG data", and "mAb data" respectively.

Figure 3

	Mean	SD
OCT	2.47E-04	9.34E-05
GG	1.20E-04	2.96E-05

Figure 4A

	Untreated		2,6ST		NEU	
	Mean	SD	Mean	SD	Mean	SD
OCT	0.3039	0.0301	0.2535	0.0311	0	0
GG	0.2943	0.019	0.2988	0.0316	0	0
_6E10	0.0847	5.73E-03	0.0819	4.26E-03	0	0
4G8a	0.2147	0.0181	0.2822	0.0287	0	0
4G8b	0.4246	0.0602	0.6965	0.011	0	0

Figure 4B

	Untreated		2,6ST		NEU	
	Mean	SD	Mean	SD	Mean	SD
OCT	0.0792	2.86E-03	0.0982	4.96E-03	-7.34E-04	0.0134
GG	0.101	8.50E-03	0.1098	6.50E-03	-7.53E-03	0.0164
_6E10	0.0474	3.16E-03	0.0573	0.0211	8.70E-03	0.0171
4G8a	0.0859	7.23E-03	0.1129	0.0115	5.79E-03	8.19E-03
4G8b	0.1698	0.0241	0.2786	4.40E-03	5.98E-03	8.45E-03

Figure 4C

	Untreated		2,6ST		NEU	
	Mean	SD	Mean	SD	Mean	SD
OCT	5.21E-03	4.04E-03	0.0187	1.67E-03	0.0606	3.90E-03
GG	0.029	0.0104	0.0312	8.46E-03	0.0762	0.0181
_6E10	0.044	0.0171	0.0411	0.0522	0.0118	0.0113
4G8a	0.0263	0.0102	0.0228	5.50E-03	0.2066	0.0343
4G8b	0.0393	0.0152	0.0195	4.71E-03	0.3375	0.056

Figure 5B

All data			IVIG data			mAb data		
CFCA	On-Chip (raw)	On-Chip(adjusted)	CFCA	On-Chip (raw)	On-Chip(adjusted)	CFCA	On-Chip (raw)	On-Chip(adjusted)
0.2826	0.0772	0.1214	0.2826	0.0772	0.1214	0.0806	0.0496	0.078
0.2755	0.1017	0.1599	0.2755	0.1017	0.1599	0.0789	0.0722	0.1135
0	8.76E-03	0.0138	0	8.76E-03	0.0138	0	-3.38E-03	-5.31E-03
0.3252	0.0813	0.1278	0.3252	0.0813	0.1278	0.0887	0.0451	0.071
0.2316	0.0947	0.1489	0.2316	0.0947	0.1489	0.0849	0.0424	0.0667
0	-0.0102	-0.0161	0	-0.0102	-0.0161	0	0.0208	0.0327
0.2809	0.107	0.1683	0.2809	0.107	0.1683	0.2275	0.0808	0.127
0.2764	0.1144	0.1799	0.2764	0.1144	0.1799	0.2619	0.121	0.1902
0	4.04E-03	6.36E-03	0	4.04E-03	6.36E-03	0	0.0116	0.0182
0.3077	0.095	0.1494	0.3077	0.095	0.1494	0.2019	0.091	0.1431
0.3211	0.1052	0.1655	0.3211	0.1052	0.1655	0.3025	0.1048	0.1647
0	-0.0191	-0.03	0	-0.0191	-0.03	0	0.0011	0.0015
0.0806	0.0496	0.078				0.4672	0.1528	0.2403
0.0789	0.0722	0.1135				0.7043	0.2755	0.4331
0	-3.38E-03	-5.31E-03				0	0.012	0.0188
0.0887	0.0451	0.071				0.382	0.1869	0.2938
0.0849	0.0424	0.0667				0.6887	0.2817	0.4429
0	0.0208	0.0327				0	-0.0006	-0.001
0.2275	0.0808	0.127						
0.2619	0.121	0.1902						
0	0.0116	0.0182						
0.2019	0.091	0.1431						
0.3025	0.1048	0.1647						
0	0.0011	0.0015						
0.4672	0.1528	0.2403						
0.7043	0.2755	0.4331						
0	0.012	0.0188						
0.382	0.1869	0.2938						
0.6887	0.2817	0.4429						
0	-0.0006	-0.001						

Figure 7

	Mean	SD
OCT	0.8763	0.169
GG	0.8587	0.1643
_6E10	-0.1454	0.1721
4G8a	0.7536	0.1905
4G8b	0.9467	0.1633

Figure 8A

SNA	Mean	SD
OCT None_None	4.08E-03	0.0665
OCT None_EndoS	0.093	0.0513
OCT 2,6ST_None	0.2006	0.0724
OCT 2,6ST_EndoS	0.0721	0.0513
OCT NEU_None	0.0518	0.0416
OCT NEU_EndoS	0.0281	0.0513
GG None_None	0.01	0.0589
GG None_EndoS	0.0385	0.0513
GG 2,6ST_None	0.2137	0.0421
GG 2,6ST_EndoS	0.0542	0.0513
GG NEU_None	0.0673	0.0264
GG NEU_EndoS	0.0298	0.0513

Figure 8B

ECL	Mean	SD
OCT None_None	-4.87E-03	0.1057
OCT None_EndoS	0.0322	0.0786
OCT 2,6ST_None	0.1974	0.1365
OCT 2,6ST_EndoS	-0.0667	0.0786
OCT NEU_None	0.3859	0.061
OCT NEU_EndoS	0.3359	0.0786
GG None_None	4.72E-03	0.0849
GG None_EndoS	-0.0798	0.0786
GG 2,6ST_None	0.2539	0.0512
GG 2,6ST_EndoS	-0.0623	0.0786
GG NEU_None	0.3297	0.0322
GG NEU_EndoS	0.3916	0.0786
